# ﻿*Synotisjinpingensis* (Asteraceae, Senecioneae), a new species with white ray florets from southeastern Yunnan, China

**DOI:** 10.3897/phytokeys.235.112230

**Published:** 2023-11-20

**Authors:** Liao-Chen Zhao, Ren Chen, Zhi-Yong Yu, Ming Tang, Qin-Er Yang

**Affiliations:** 1 College of Forestry, Jiangxi Agricultural University, Nanchang 330045, Jiangxi, China; 2 Key Laboratory of Plant Resources Conservation and Sustainable Utilization, South China Botanical Garden, Chinese Academy of Sciences, Guangzhou 510650, Guangdong, China; 3 Center of Conservation Biology, Core Botanical Gardens, South China Botanical Garden, Chinese Academy of Sciences, Guangzhou 510650, Guangdong, China; 4 Administrative Bureau of Fenshuiling National Nature Reserve, Jinping 661500, China

**Keywords:** Compositae, floral micromorphology, ITS sequence data, taxonomy

## Abstract

*Synotisjinpingensis* (Asteraceae, Senecioneae), a new species from Jinping county in southeastern Yunnan province, China, is described and illustrated. This species is distinguished by having white ray florets in the genus *Synotis*, in which only species with yellow ray florets have been hitherto known. In habit and leaf shape *S.jinpingensis* is most closely similar to *S.duclouxii*, a species occurring in southwestern Guizhou, southern Sichuan and northeastern Yunnan, China, but differs, in addition to the color of ray florets, by having fewer lateral veins of leaves, obviously longer bracts of calyculus, and larger phyllaries. The membership of the new species within *Synotis* is strongly corroborated by evidence from floral micromorphology and phylogenetic analyses based on ITS sequence data. Color photographs of living plants, a distribution map, and provisional IUCN status of *S.jinpingensis* are provided.

## ﻿Introduction

*Synotis* (Clarke) C. Jeffrey & Y.L. Chen (Asteraceae, Senecioneae), was segregated from *Senecio* L. based on its sub-shrubby habit and anther bases with sterile tailed auricles. *Synotis* is endemic to the Sino-Himalayan region, except for two species occurring in northwestern China and Kyrgyzstan (Jeffrey and Chen 1984; Li et al. 2018). Sixty species are currently recognized in the genus, with 48 recorded in China (Jeffrey and Chen 1984; Chen 1999; Chen et al. 2011; Tang et al. 2013a, b, c, 2014, 2017, 2022; Tong et al. 2017; Li et al. 2020; Liu et al. 2020, 2021; Tang and Chen 2021; Zhang et al. 2021; Fan et al. 2022a, b).

**Figure 1.**
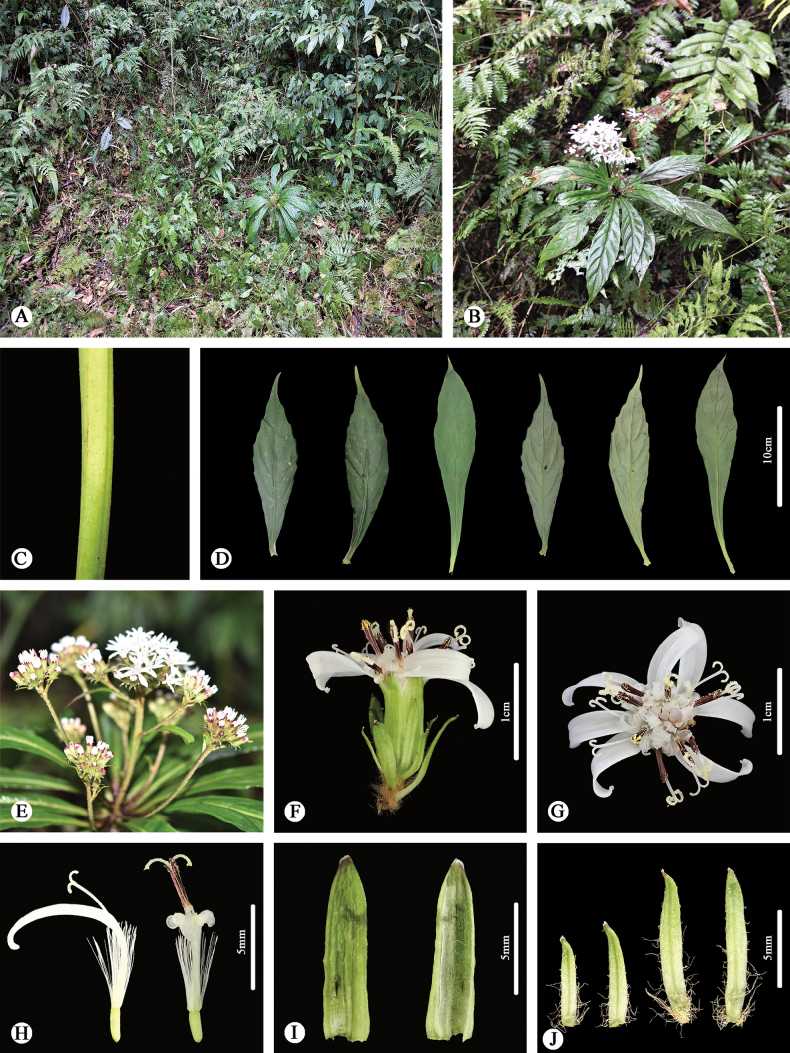
*Synotisjinpingensis* in the wild (Jinping, Yunnan, China) **A** habitat **B** habit **C** portion of stem **D** leaves (left three: adaxial side; right three: abaxial side) **E** synflorescence **F** capitulum (side view) **G** capitulum (top view) **H** florets (left: ray floret; right: disk floret) **I** phyllaries (left: adaxial side; right: abaxial side) **J** bracts of calyculus.

**Figure 2.**
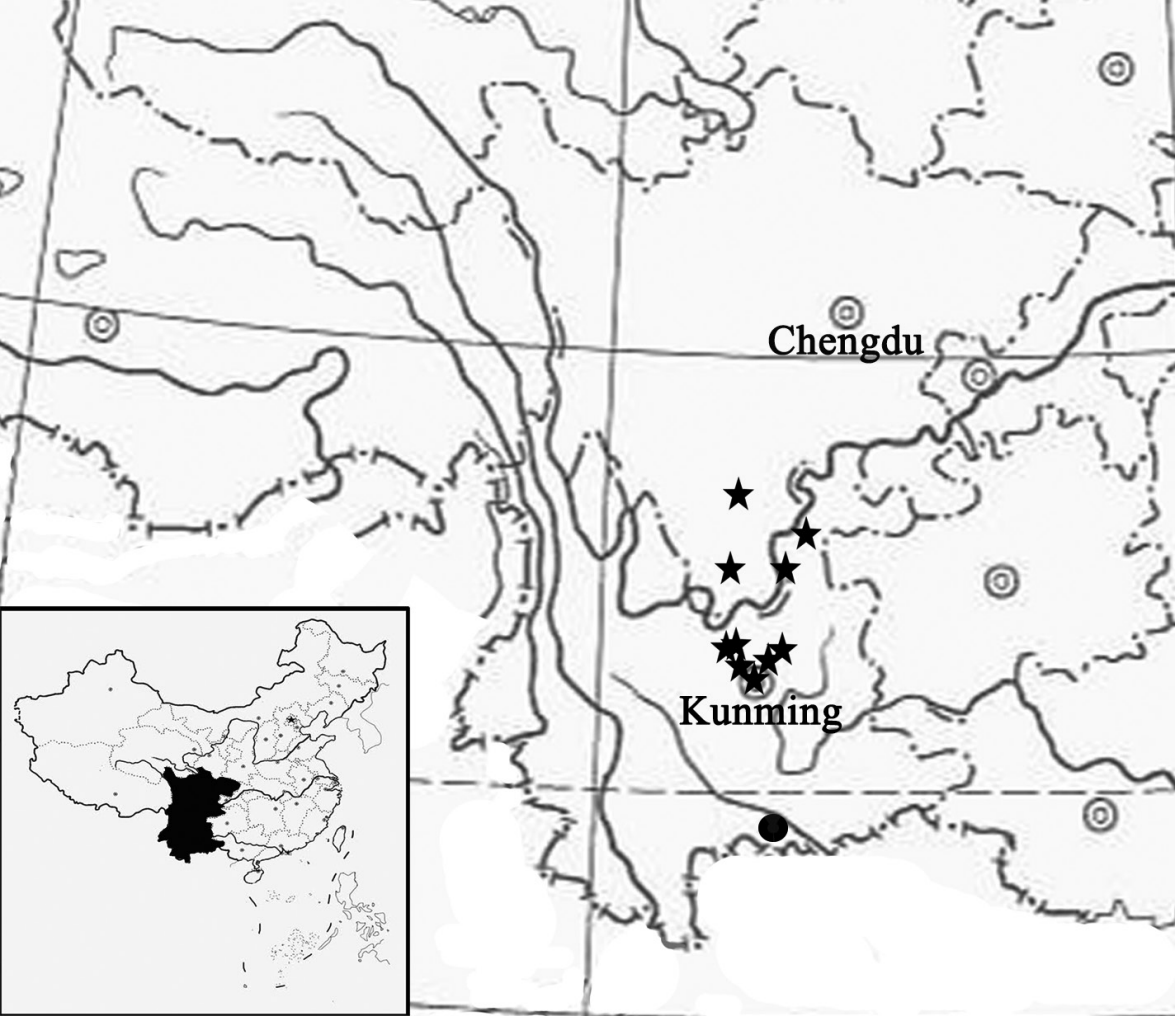
Distribution map of *Synotisjinpingensis* (black dot) and *S.duclouxii* (black star).

During a botanical trip in March 2022 to southeastern Yunnan, China, we discovered an unusual population of *Synotis* with white ray florets (Fig. 1) in the Fenshuiling National Nature Reserve in Jinping county (Fig. 2). All the previously known species in *Synotis* have yellow ray florets. The habit and the obovate-lanceolate or elliptic leaves of the plants are most closely similar to those of *S.duclouxii*, a species occurring in southwestern Guizhou, southern Sichuan, and northeastern Yunnan, China (Liu et al. 2020) (Fig. 2), but differ by having white florets and several other morphological characters, including fewer nerves on the leaves and longer bracts of calyculus. We conducted floral micromorphological studies and performed phylogenetic analyses based on ITS sequence data to further confirm the membership of the new species within the genus *Synotis*. We therefore determine that the population in question represents a hitherto undescribed species, which we describe below.

## ﻿Material and methods

### ﻿Gross morphology

We conducted careful observations of living plants in a population of the new species in Jinping county in southeastern Yunnan, China. All the major morphological characters, including the habit, the shape and size of leaves, involucres, ray and disk florets, and the type of synflorescence, which were observed and photographed On living plants by digital camera (OLYMPUS TG-6, Tokyo).

### ﻿Floral micromorphology

For the study of three floral micromorphological characters of the new species (voucher: *Z. Y. Yu et al. JXAU 01*, JXAU), including papillae on style arms, filament collar, and anther endothecial cell thickenings in stamens [(for all these three micromorphological characters were considered taxonomic significance in the *Synotis* reported by Jeffrey and Chen (1984) and Tang (2014)], we followed the method of Tang et al. (2014), and all these characters were observed and photographed under microscope (ZEISS AXIO IMAGER A2M, Germany).

### ﻿Phylogenetic analyses

Based on the results of previous phylogenetic analyses of the tribe Senecioneae (Pelser et al. 2002, 2003, 2007, 2010), we selected 82 samples representing 79 species in 38 genera within Senecioneae for our analyses. These included 30 species of *Senecio* and 13 species in *Synotis*. *Abrotanellaemarginata* Cass. (subtribe Abrotanellinae) was selected as a root, according to the results of Pelser et al. (2007, 2010). We generated new sequences for our new species and its putative close ally *S.duclouxii*, and another species *S.cavaleriei* (H. Lév.) C. Jeffrey & Y.L. Chen, while the sequences of other species were retrieved from GenBank. Voucher information and GenBank accession numbers for the material used in this study are given in Appendix 1.

Total genomic DNA of *Synotiscavaleriei*, *S.duclouxii* and our new species were extracted from silica gel-dried leaves using the modified CTAB method of Doyle and Doyle (1987). The nuclear regions (ITS) were sequenced, with the primer pairs ITS4 and ITS5 (White et al. 1990). Amplification and sequencing reactions followed Tang (2014) and Ren et al. (2017).

All sequences were aligned with MAFFT 7.450 (Katoh and Standley 2013). ModelFinder (Kalyaanamoorthy et al. 2017) was used to select the best-fit model using BIC criterion. Maximum Likelihood (ML) analysis was generated by IQ-TREE 2.1.3 (Nguyen et al. 2015), with 20 000 ultrafast bootstraps, under GTR+F+I+I+R3 model by ModelFinder. Bayesian Inference (BI) (Ronquist et al. 2012) analysis was carried out by MrBayes 3.2.6 (Ronquist et al. 2012), under GTR+F+I+G4 model, with 3 000 000 generations, sampling every 1 000 generations to ensure the convergence (average deviation of split frequencies less than 0.01 and the effective sample sizes over 200), in which the first 25% of sampled data treated were discarded as burn-in and the remaining trees were used to estimate the posterior probabilities (PP). Bootstrap percentage (MLBS) values ≥ 70 (Hillis and Bull 1993) and PP values ≥ 0.95 were regarded as strong support.

## ﻿Results and discussion

### ﻿Gross morphology

As shown in Figs 1, 2, our new species has a habit typical of Synotisser.Synotis (Jeffrey and Chen 1984), with the leaves clustered at the bottom of the synflorescence, indicating that this species should belong to this series. It is readily distinguishable from all other species within the genus by its white ray florets. From its putative closest ally, *S.duclouxii*, the new species differs additionally by having fewer nerves on the leaves (10–14 vs. 18–20), longer bracts of calyculus (6–8 mm vs. 1–3 mm) and larger phyllaries (8–10 mm long, 2–3 mm broad vs. 5–7 mm long, 1–2 mm broad) [see Liu et al. (2020) for morphological characters of *S.duclouxii*].

### ﻿Floral micromorphology

**Figure 3.**
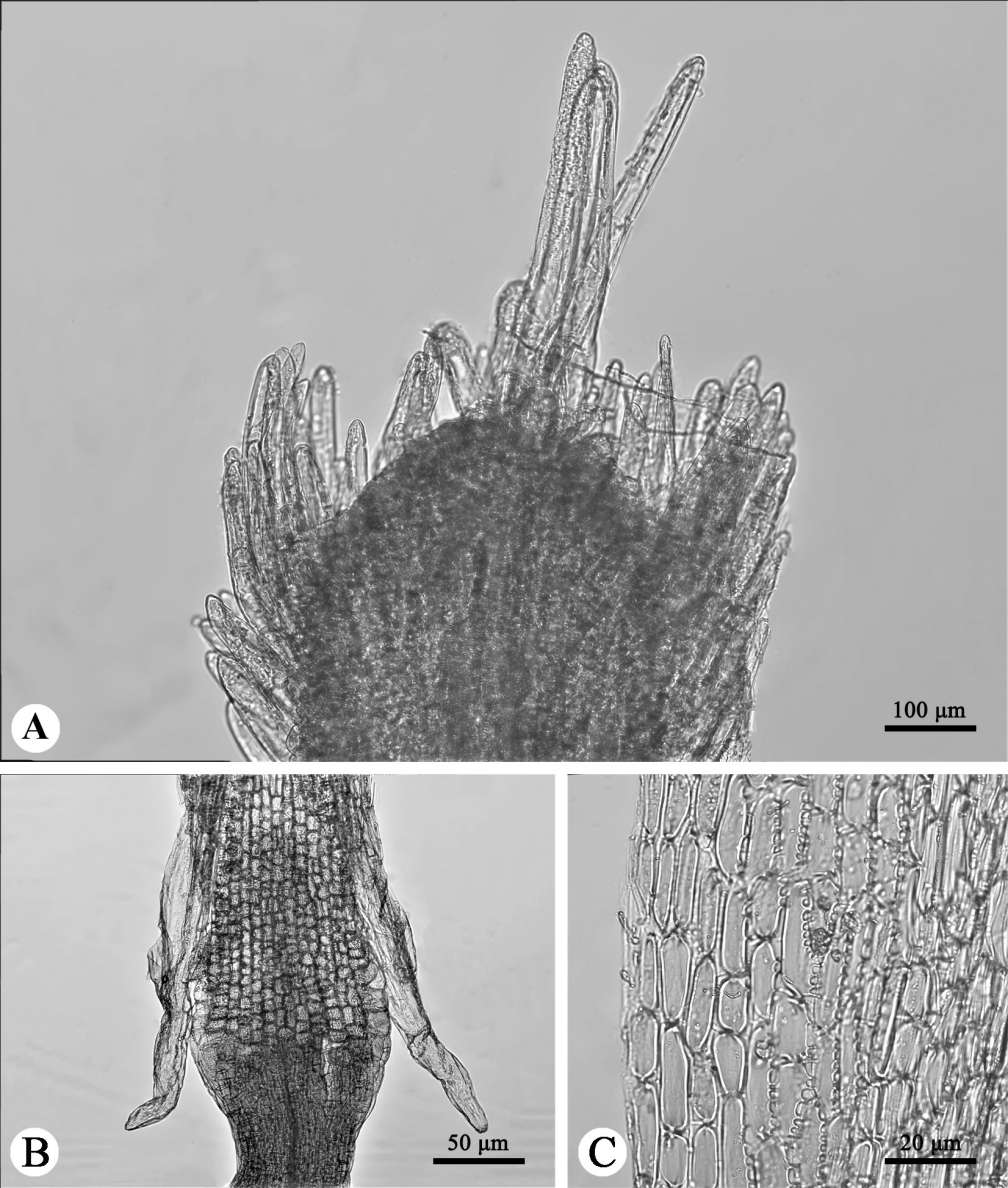
Three micromorphological characters of *Synotisjinpingensis***A** papillae on style arms **B** anther collar and anther tails **C** anther endothecial cell wall thickenings.

The central tuft of papillae on the style arms of our new species is prominent, much longer than laterals (Fig. 3A). The anther collars are balusterform (Fig. 3B), the anther tails are ca. 1.5 times as long as antheropodia (Fig. 3B), and the endothecial cell wall thickenings are radial (Fig. 3C). All these characters match well those reported previously in *Synotis* (Jeffrey and Chen 1984; Tang et al. 2013a, b, 2014; Tang 2014; Li et al. 2018) and further confirm the generic affiliation of our new species.

### ﻿Phylogenetic analyses

Our ML and BI analyses produce almost identical topologies, and they are also consistent with the results of previous studies (Pelser et al. 2007, 2010; Tang et al. 2014; Tong et al. 2017; Li et al. 2018). As shown in the ML tree (Fig. 5), all the sampled species of *Synotis* form a strongly supported clade (MLBS/PP = 99%/1.00) in subtribe Senecioninae and our new species is deeply nested within this clade. The membership of our new species within *Synotis* is thus strongly corroborated by ITS sequence data.

### ﻿Taxonomic treatment

#### ﻿ Synotis jinpingensis

Taxon classificationPlantaeAsteralesAsteraceae

M.Tang, Z.Y.Yu & Q.E.Yang sp. nov.

54325AEC-158F-5B40-9673-A933DA627C77

urn:lsid:ipni.org:names:77331168-1

Figs 1 , 4

##### Type.

**Figure 4.**
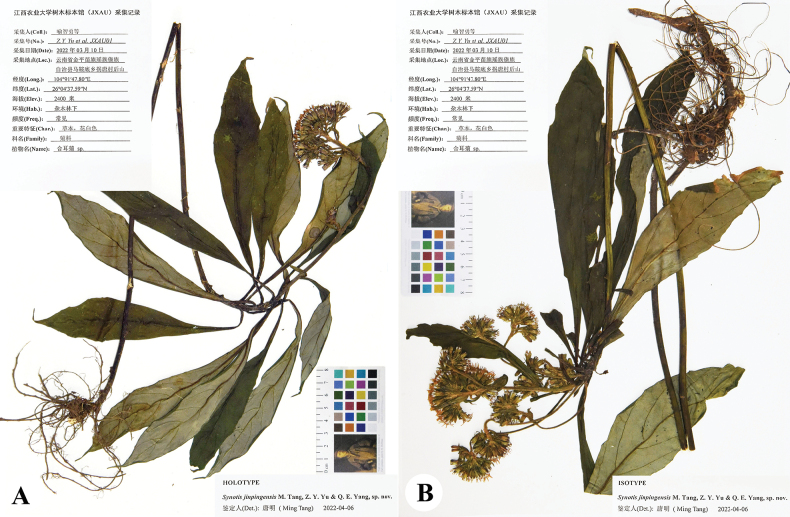
Holotype (**A**) and isotype (**B**) sheets of *Synotisjinpingensis*.

China. Yunnan province: Jinping county, Fenshuiling National Nature Reserve, Guaitang village, in mixed forests, alt. ca. 2400 m, 22°45′36.87″N, 103°28′4.65″E, 30 March 2022, *Z.Y. Yu et al. JXAU 01* (holotype: JXAU; isotypes: IBSC, JXAU). Fig. 4.

##### Diagnosis.

*Synotisjinpingensis* is most closely similar to *S.duclouxii* in habit and leaf shape, but differs by having white (vs. yellow) ray florets, fewer nerves on the leaves (10–14 vs. 18–20), longer bracts of calyculus (6–8 mm vs. 1–3 mm), and larger phyllaries (8–10 mm long, 2–3 mm broad vs. 5–7 mm long, 1– 2 mm broad).

**Figure 5.**
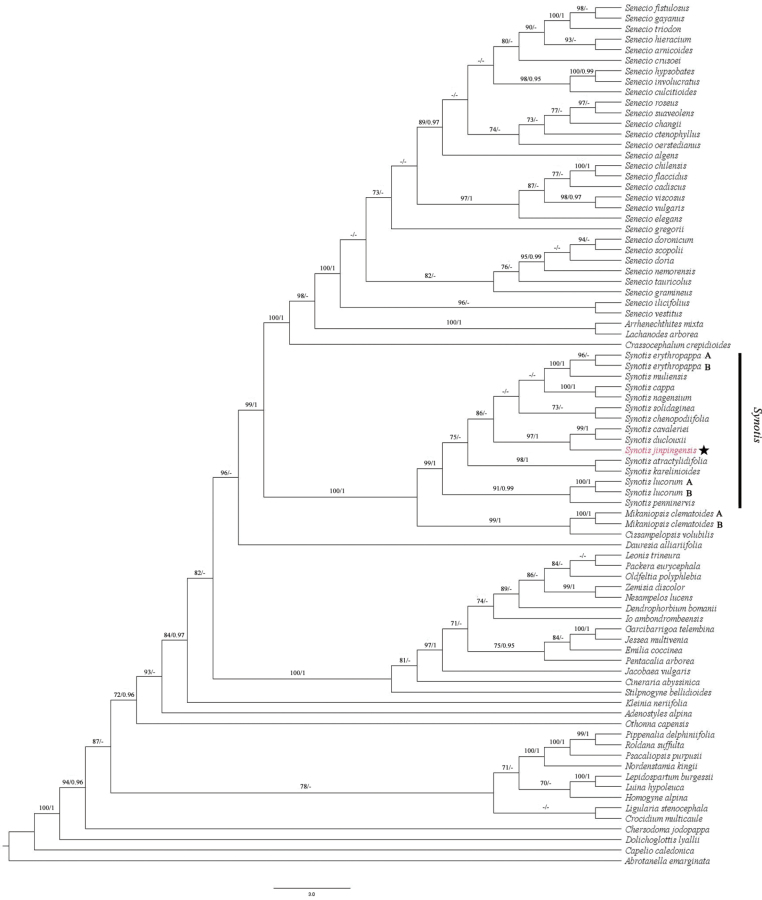
The maximum likelihood tree of tribe Senecioneae based on ITS dataset, with *Synotisjinpingensis* highlighted in red font. Bootstrap values (MLBS) and posterior probabilities (PP) are indicated above the branches. Dashes (-) indicate MLBS < 70% or PP < 0.95.

##### Description.

Perennial herbs, erect, rhizomatous. Rhizome thick, horizontal. Vegetative stems solitary, simple, rarely branched, erect, 50–100 cm tall, median and lower parts subglabrous, upper part often densely yellowish setulose. Flowering stems solitary, erect, scapiform, 15–30 cm tall, few-branched, fulvous tomentose. Leaves always aggregate at base of fertile shoot; petioles 1–1.5 cm long; blades obovate-lanceolate or elliptic, 12–18 cm long, 2.5–4 cm broad, papyraceous, abaxially glabrous or subglabrous, adaxially sparsely pubescent, pinnately veined, lateral veins 10–14, arcuate-ascending, base cuneate, margin shallowly sinuate-apiculate, apex acute-acuminate. Stem leaves on reproductive shoots few, narrowly lanceolate, remote, much smaller. Capitula radiate, numerous, arranged in an attenuate broadly paniculoid corymb; peduncles 3–5 mm long, fulvous tomentose, 1- or 2-bracteate; bracts below capitula linear, 5–20 mm long. Involucres cylindric-campanulate, 8–10 mm long, 2–3 mm broad, with 7–10 subulate bracteoles at base, bracts of calyculus linear, 6–8 mm long, green, apically purple; phyllaries usually 7 or 8, occasionally 5 or 6, oblong, 2–3 mm broad, green, herbaceous, glabrous, apically acute, purple. Ray florets 7 or 8; corolla tube 3.5–4.5 mm long, glabrous; lamina white, oblong-lanceolate, 6–9 mm long, 1.5–2.5 mm broad, 3–4-veined, apically obtuse, 3-denticulate. Disk florets 8–12, white; corolla 4–5 mm long, with ca. 4.5 mm long tube and funnelform limb; lobes ovate-oblong, 3–3.5 mm long, apically acute. Anthers ca. 3 mm, anther tails ca. 1.5 times as long as antheropodia; appendages ovate-oblong; antheropodia slightly dilated at base. Style branches ca. 2 mm long, fringed with long fine papillae, the central tuft prominent, much longer than laterals. Achenes 1.8–2 mm, glabrous. Pappus white, 6–7.5 mm long.

##### Phenology.

Flowering from March to April; fruiting from April to July.

##### Etymology.

The species is named after its type locality, i.e., Jinping county in southeastern Yunnan province, China.

##### Distribution and habitat.

*Synotisjinpingensis* is currently known from its type locality, i.e., Jinping county in southeastern Yunnan province, China (Fig. 2). It grows in mixed forests at an altitude of ca. 2400 m above sea level.

##### Conservation status.

*Synotisjinpingensis* seems currently known only from its type locality. Four small populations of this species, each with ca. 100 individuals, have been discovered there. The habitat of *S.jinpingensis* is now well preserved. The discovery of further populations of this species is to be expected as botanical exploration of southeastern Yunnan proceeds. According to the IUCN Red List Categories and Criteria (IUCN 2022), the new species may better be categorized as Data Deficient (DD).

## Supplementary Material

XML Treatment for Synotis jinpingensis

## ﻿Additional information

### Author ORCIDs

Liao-Chen Zhao https://orcid.org/0000-0002-6300-7301

Ren Chen https://orcid.org/0000-0002-0910-9737

Zhi-Yong Yu https://orcid.org/0000-0001-8276-5901

Ming Tang https://orcid.org/0000-0001-8419-0996

Qin-Er Yang https://orcid.org/0000-0002-6261-0731

### Data availability

All of the data that support the findings of this study are available in the main text.

## ﻿Appendix 1

**Table A1.** Voucher information and GenBank accession of species used in our study.

GenBank	Species	Voucher information	Herbarium
MH371137	*Synotisatractylidifolia* (Y. Ling) C. Jeffrey & Y. L. Chen	M. Tang & L. Wang 361	IBSC
EF538402	*Synotiscappa* (Buch.-Ham. ex D. Don) C. Jeffrey & Y. L. Chen	Tessier-Yandell 86	IBSC
KJ851593	*Synotischenopodiifolia* (DC.) M. Tang et al.	M. Tang & C. Ren 532	IBSC
KU696133	*Synotiserythropappa* (Bureau & Franch.) C. Jeffrey & Y. L. Chen **A**	C. Ren 150	IBSC
MH117782	*Synotiserythropappa* (Bureau & Franch.) C. Jeffrey & Y. L. Chen **B**	YLDP026D	IBSC
MH371138	*Synotiskarelinioides* (C. Winkl.) C. Ren et al.	H.M. Li & Q.G. Mao 325	IBSC
AY723255	*Synotislucorum* (Franch.) C. Jeffrey & Y. L. Chen **A**	J.Q. Liu 2177	IBSC
KU696134	*Synotislucorum* (Franch.) C. Jeffrey & Y. L. Chen **B**	Q.E. Yang et al. 3128	IBSC
KU696135	*Synotismuliensis* Y. L. Chen	C. Ren 148	IBSC
AF459922	*Synotisnagensium* (C. B. Clarke) C. Jeffrey & Y. L. Chen	B. Bartholomew et al. 1991	IBSC
KY347902	*Synotispenninervis* (H. Koyama) T.J. Tong et al.	M. Tang & C. Ren 626	IBSC
KX549951	*Synotissolidaginea* (Hand.-Mazz.) C. Jeffrey & Y. L. Chen	2015XZ001-B	IBSC
–	*Synotisduclouxii* (Dunn) C. Jeffrey & Y. L. Chen	C. Ren & L.Y. Wang 485	IBSC
–	*Synotiscavaleriei* (H. Lév.) C. Jeffrey & Y. L. Chen	L.Y. Wang 72	IBSC
–	*Synotisjinpingensis* M. Tang et al.	Y. Yu et al. JXAU01	JXAU
EF538296	*Senecioalgens* Wedd.	S.G. Beck 2879	S
EF538298	*Senecioarnicoides* Hook. & Arn.	O. Zoellner 3474	L
GU818506	*Seneciocadiscus* B. Nord. & Pelser	Rourke 1118	S
KU499905	*Seneciochangii* C. Ren & Q. E. Yang	C. Ren et al. WL146	IBSC
EF538313	*Seneciochilensis* Less.	O. Zollner 2958	S
EF538290	*Seneciocrusoei* Pelser	T. Stussey 6560	H
EF538322	*Senecioctenophyllus* Phil.	O. Zoellner 3959	U
EF538312	*Senecioculcitioides* Wedd.	B. Ollgaard & H. Balslev 8822	CHR
AF459946	*Seneciodoria* L.	P.B. Pelser cult. 129	WIS
JX895355	*Seneciodoronicum* (L.) L.	J. Calvo 4000	WIS
GU818642	*Senecioelegans* L.	Cron & Goodman 687	–
EF538335	*Seneciofistulosus* Poepp. ex Less.	S.G. Beck & M. Liberman 9672	MJG
EF538336	*Senecioflaccidus* Less.	J. Thuret s.n.	MJG
GU818649	*Seneciogayanus* DC.	M. Rosas 2157	–
GU818650	*Seneciogramineus* Harv.	F.K. Hoener 2104	–
GU818651	*Seneciogregorii* F. Muell.	D.E. Albrecht 7091	–
GU818652	*Seneciohieracium* J. Rémy	M. Baeza & L. Finot 3695	–
EF538348	*Seneciohypsobates* Wedd	B. Ollgaard & H. Balslev 9863	U
GU818662	*Senecioilicifolius* L.	Cron & Goodman 686	
EF538150	*Senecioinvolucratus* (Kunth) DC.	B. Nordenstam 9438	L
AF459937	*Senecionemorensis* L.	P.B. Pelser cult.102	L
EF538362	*Seneciooerstedianus* Benth.	B. Nordenstam 9160	S
EF538373	*Senecioroseus* Sch. Bip.	J. Garcia P. 250	U
JX895384	*Senecioscopolii* Hoppe & Hornsch. ex Bluff & Fingerh.	J. Calvo 4715	–
EF538222	*Seneciosuaveolens* (L.) Elliott	D.C. Dister s.n.	MJG
GU817570	*Seneciotauricolus* V. A. Matthews	Budak et al. 1735	–
GU818707	*Seneciotriodon* Phil.	F. Luebert & S. Teillier 2266	–
GU818708	*Seneciovestitus* P. J. Bergius	W. Greuter 21766	–
AF459925	*Senecioviscosus* L.	P.B. Pelser 300	TEX
AF459924	*Seneciovulgaris* L.	P.B. Pelser cult. 188	CHR
GU818721	*Stilpnogynebellidioides* DC.	P. Goldblatt & L. Porter 11729	–
EF538416	*Zemisiadiscolor* (Sw.) B. Nord.	G.L. Webster et al. 8420	S
EF538143	*Abrotanellaemarginata* (Gaudich.) Cass.	R.N.P. Goodall & J. Wood 3352	MU
EF538146	*Adenostylesalpina* Bluff & Fingerh.	C.H. Uhink 98-189a	MJG
EF538156	*Arrhenechthitesmixta* (A. Rich.) Belcher	M.E. Lawrence 1308	S
GU818508	*Capeliocaledonica* B. Nord.	B. Nordenstam 9644	–
EF538167	*Chersodomajodopappa* (Sch. Bip.) Cabrera	I. Hensen 2617	S
GU818512	*Cinerariaabyssinica* Sch. Bip. ex A. Rich.	P.B. Pelser cult. 208	L
EF538172	*Cissampelopsisvolubilis* (Blume) Miq.	Carvalho 3175	US
AF459968	*Crassocephalumcrepidioides* (Benth.) S. Moore	P.B. Pelser cult. 354	S
GU818541	*Crocidiummulticaule* Hook.	B. Bartholomew 5749	–
AF457413	*Dauresiaalliariifolia* (O. Hoffm.) B. Nord. & Pelser	Mueller & Tilson 907	–
EF538181	*Dendrophorbiumbomanii* (R. E. Fr.) C. Jeffrey	M. Dematteis & G. Seijo 722	MU
GU818546	*Dolichoglottislyallii* (Hook. f.) B. Nord.	A. Strid 22172	–
AF459966	*Emiliacoccinea* (Sims) G. Don	P.B. Pelser cult. 126	MJG
EF538211	*Garcibarrigoatelembina* (Cuatrec.) Cuatrec.	L. Holm-Nielsen et al. 6211	S
KU570815	*Homogynealpina* Cass.	Comes 11	MJG
GU818559	*Ioambondrombeensis* (Humbert) B. Nord.	S.T. Malcomber et al. 1380	–
AF459941	*Jacobaeavulgaris* Gaertn.	P.B. Pelser cult. 6	S
EF538246	*Jesseamultivenia* (Benth.) H. Rob. & Cuatrec.	B. Nordenstam 9161	K
GU818568	*Kleinianeriifolia* Haw.	P.B. Pelser cult. 216	–
GU818574	*Lachanodesarborea* (Roxb.) B. Nord.	R. Cairns-Wicks s.n.	–
EF538249	*Leonis trineura* (Griseb.) B. Nord.	Smith et al. 3238	L
EF538250	*Lepidospartumburgessii* B.L. Turner	R.D. Worthington 12382	L
AF459961	*Ligulariastenocephala* (Maxim.) Matsum. & Koidz.	R.R. Kowal 3092	WIS
GU818593	*Luinahypoleuca* Benth.	W. Greuter 17706	–
GU818595	*Mikaniopsisclematoides* (Sch. Bip. ex A. Rich.) Milne-Redh. **A**	W.J.J.O. de Wilde & B.E.E. de Wilde-Duyfjes 9006	–
GU817581	*Mikaniopsisclematoides* (Sch. Bip. ex A. Rich.) Milne-Redh. **B**	I. Friis et al. 499	–
EF538266	*Nesampeloslucens* (Poir.) B. Nord.	Zanoni 45570	JBSD
EF538267	*Nordenstamiakingii* (H. Rob. & Cuatrec.) B. Nord.	B. Stahl 5572A	S
EF538271	*Oldfeltiapolyphlebia* (Griseb.) B. Nord. & Lundin	B. Nordenstam & R. Lundin 340	S
AF459960	*Othonnacapensis* L. H. Bailey	P.B. Pelser cult. 106	S
GU818608	*Packeraeurycephala* (Torr. & A. Gray) W.A. Weber & Á. Löve	M.A. Vincent 8581	MU
EF538283	*Pentacaliaarborea* (Kunth) H. Rob. & Cuatrec.	B. Ollgaard & H. Balslev 8298	MU
GU818627	*Pippenaliadelphiniifolia* (Rydb.) McVaugh	Spellenberg & Bacon 11048	–
GU818629	*Psacaliopsispurpusii* (Greenm. ex Brandegee) H. Rob. & Brettell	Panero et al. 2607	–
GU818631	*Roldanasuffulta* (Greenm.) H. Rob. & Brettell	Rzedowski 36569	–
